# Corals and sponges are hotspots of reactive oxygen species in the deep sea

**DOI:** 10.1093/pnasnexus/pgad398

**Published:** 2023-11-15

**Authors:** Lina Taenzer, Scott D Wankel, Jason Kapit, William A Pardis, Santiago Herrera, Steven Auscavitch, Kalina C Grabb, Erik Cordes, Colleen M Hansel

**Affiliations:** Marine Chemistry and Geochemistry, Woods Hole Oceanographic Institution, Woods Hole, MA 02543, USA; Earth, Atmospheric and Planetary Sciences, Massachusetts Institute of Technology, Cambridge, MA 02139, USA; Marine Chemistry and Geochemistry, Woods Hole Oceanographic Institution, Woods Hole, MA 02543, USA; Applied Ocean Physics and Engineering, Woods Hole Oceanographic Institution, Woods Hole, MA 02543, USA; Applied Ocean Physics and Engineering, Woods Hole Oceanographic Institution, Woods Hole, MA 02543, USA; Department of Biological Sciences, Lehigh University, Bethlehem, PA 18015, USA; Department of Biology, Boston University, Boston, MA 02215, USA; Marine Chemistry and Geochemistry, Woods Hole Oceanographic Institution, Woods Hole, MA 02543, USA; Earth, Atmospheric and Planetary Sciences, Massachusetts Institute of Technology, Cambridge, MA 02139, USA; Department of Biology, Temple University, Philadelphia, PA 19122, USA; Marine Chemistry and Geochemistry, Woods Hole Oceanographic Institution, Woods Hole, MA 02543, USA

**Keywords:** deep-sea coral, reactive oxygen species, submersible sensor, superoxide, NOX

## Abstract

Reactive oxygen species (ROS) are central to diverse biological processes through which organisms respond to and interact with their surroundings. Yet, a lack of direct measurements limits our understanding of the distribution of ROS in the ocean. Using a recently developed in situ sensor, we show that deep-sea corals and sponges produce the ROS superoxide, revealing that benthic organisms can be sources and hotspots of ROS production in these environments. These findings confirm previous contentions that extracellular superoxide production by corals can be independent of the activity of photosynthetic symbionts. The discovery of deep-sea corals and sponges as sources of ROS has implications for the physiology and ecology of benthic organisms and introduces a previously overlooked suite of redox reactants at depth.

## Introduction

Reactive oxygen species (ROS) are a group of oxygen-containing molecules that influence myriad processes, from organismal growth to the geochemical cycling of redox-active metals and organic matter (1). Superoxide (O_2_^•−^) is one of the most relevant ROS in marine systems. It is produced biotically through extracellular enzymatic catalysis by aerobic organisms and abiotically through photooxidation of dissolved organic matter and oxidation of reduced metals by oxygen (2), among other processes.

The short half-life (seconds to minutes) of superoxide in seawater precludes its measurement within collected seawater samples. Thus, previous estimates of in situ concentrations and production rates were extrapolated from shipboard incubations and decay kinetics in filtered waters. These studies revealed that a substantial proportion of superoxide production within the marine water column was particle-associated, presumably microbial (3, 4). Particle-associated production has been corroborated by laboratory-based studies, demonstrating extracellular superoxide production by phytoplankton and heterotrophic bacteria through light-independent and light-dependent pathways (5, 6). Recently, in situ quantification of superoxide within shallow coastal waters was made possible by using a handheld submersible sensor (DISCO), revealing elevated superoxide concentrations within tropical reef waters and at the surfaces of some coral species (7).

Based on the discoveries of superoxide production via biological light-independent reactions, we predicted that measurable superoxide may also be produced in deep-sea environments. To test this hypothesis, we designed and constructed a deep-sea chemiluminescent sensor (SOLARIS) similar in design to DISCO but capable of in situ calibration and operation to 4,000 m. SOLARIS was initially used to characterize the waters of coastal California, demonstrating steady-state concentrations over a 600-m deep-water column, well below the chlorophyll maximum (8). Similar to shallow reef observations, we further predicted that benthic macrofauna are sources of ROS to the deep sea. To test this hypothesis, we deployed SOLARIS on two dives using the human-occupied vehicle *Alvin* off the coast of California. In this study, we show the first measurements of superoxide associated with deep-sea organisms revealing corals and sponges as sources of ROS.

## Results and discussion

Superoxide concentrations measured using SOLARIS in bottom waters at Davidson Seamount (1,270 m, 10 µM oxygen, 4°C) and the Channel Islands (330 m, 30 µM oxygen, 7.8°C) ranged between 0.05 and 0.13 nM. At these depths, both sites exhibited negligible fluorescence and light. Surface water superoxide levels at these sites (∼8 to 16 nM) (8) were similar to those previously found in the surface waters of Cuba using DISCO (7). In contrast to these surface sunlit waters, superoxide generation in the aphotic waters of the deep sea is restricted to light-independent biological pathways and abiotic reaction of oxygen with reduced dissolved orangic carbon and metals. Thus, higher superoxide concentrations in the near-surface environment relative to deep waters likely reflect additional production pathways via light-dependent microbial and (photo)chemical reactions. The lower abundance and metabolic rates of microbes in deep, cold waters also likely contribute to lower overall superoxide concentrations.

**Fig. 1.**
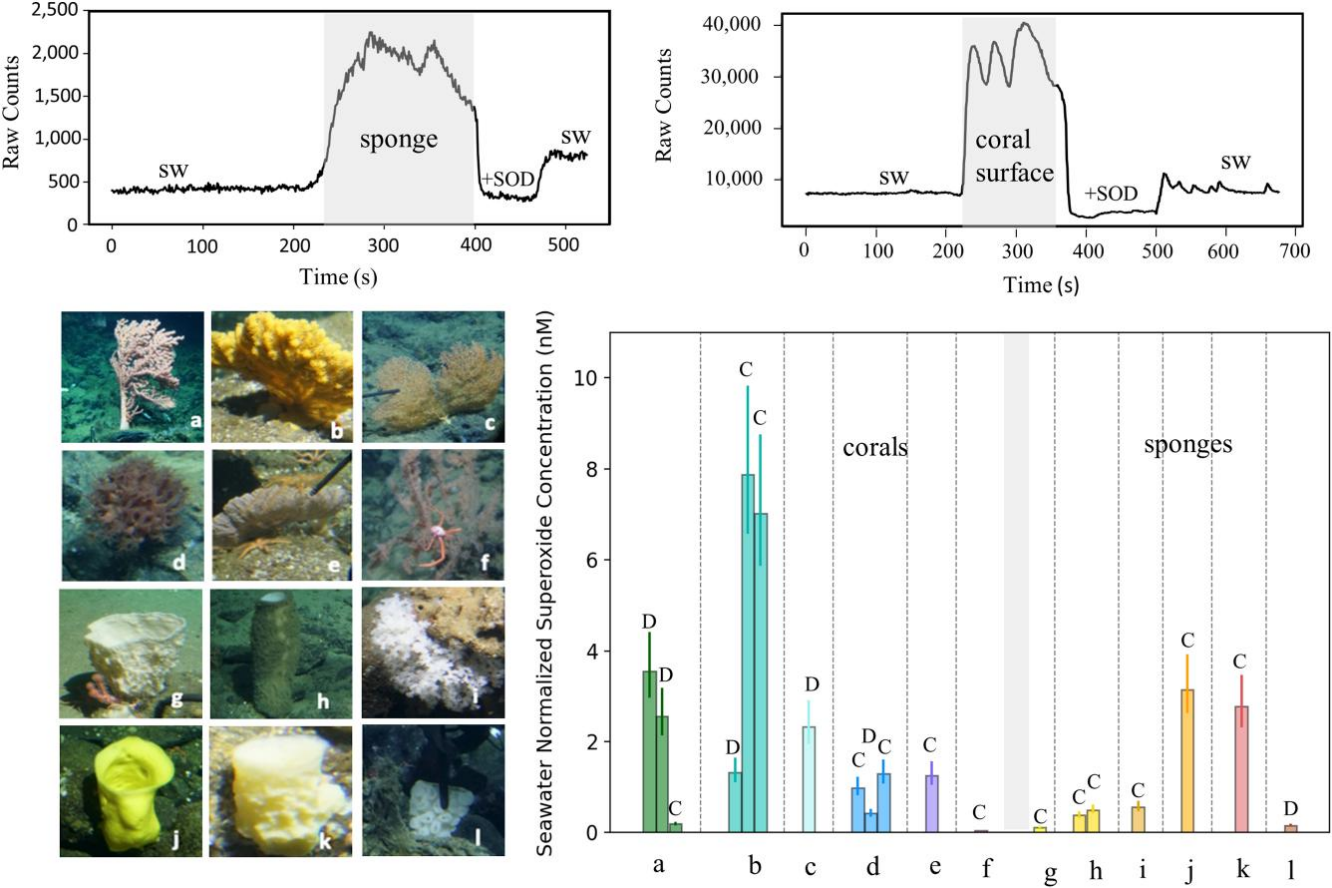
Top: Traces of the signal seen when measuring inside the exhalent osculum of the sponge *Acanthascus* sp. (left) and within a few centimeters of the surface of an *Acanthogorgia* sp. coral (right). Bottom: Comparison of the maximum superoxide concentration above background seawater detected near the surfaces of the octocorals a) *Paragorgia pacifica*, b) *Acanthogorgia* sp., c) *Swiftia* sp., d) *Anthomastus* sp., and e) *Parastenella ramosa*; the octocoral f) *Lillipathes* sp. and the sponges g) *Amphilectus* sp., h) *Acanthascus* sp., i) an unknown species of the family Farreidae, j) *Staurocalyptus* sp., k) an unknown sponge species, and l) an unknown hexactinellid species. Measurements were made during HOV *Alvin* dives near Davidson Seamount (D) and the Channel Islands (C). Each bar represents the average signal of superoxide, and the error bar reflects uncertainty in the calibration factor used to convert the raw signal of the photomultiplier tube to superoxide concentration.

We measured superoxide associated with 12 corals across 6 species and 7 sponges across 6 species over two 8-h *Alvin* dives at the study sites (Figure 1). For most corals, repositioning the SOLARIS wand from the background seawater (>1 m away from the organism) to within a few centimeters of the coral surface rapidly increased chemiluminescent counts. We eliminated these counts by adding the superoxide scavenger superoxide dismutase, thus demonstrating superoxide signal specificity (Figure 1). For sponges, there was no change in the chemiluminescent signal between the background seawater and the exterior of the sponge. Still, signals increased when we placed the wand within the exhalent osculum. Of the six coral species, only *Lillipathes* sp. did not exhibit elevated superoxide concentrations (Table 1). *Lillipathes* sp. was the only black coral species (class Hexacorallia) measured, while the other five species were octocorals (class Octocorallia). The highest signals, reaching 7.9 nM, were associated with an *Acanthogorgia* sp., representing an 80-fold increase over background seawater. Superoxide within the sponge exhalent oscula ranged from 0.1 to 3.1 nM above background seawater. These data clearly indicate that corals and sponges can be net producers and sources of superoxide to the deep sea.

**Table 1.** Average extracellular superoxide (bold) concentrations measured near the surfaces of various species of deep-sea corals and sponges using SOLARIS.

Organism	Species	Location	Concentration (nM) (uncertainty range)
Coral	*Paragorgia arborea*	Davidson Seamount	**3.52** (2.94–4.39)
		Davidson Seamount	**2.55** (2.13–3.18)
		Channel Islands	**0.19** (0.16–0.24)
	*Acanthogorgia* sp.	Davidson Seamount	**1.32** (0.99–1.65)
		Channel Islands	**7.88** (6.57–9.83)
		Channel Islands	**7.02** (5.86–8.76)
	*Swiftia* sp.	Davidson Seamount	**2.33** (1.94–2.91)
	*Anthomastus* sp.	Channel Islands	**0.98** (0.82–1.22)
		Davidson Seamount	**0.42** (0.35–0.52)
		Channel Islands	**1.29** (1.08–1.61)
	*Parastenella ramosa*	Channel Islands	**1.26** (1.05–1.57)
	*Lillipathes* sp.	Channel Islands	**0.05** (0.04–0.06)
Sponge	*Amphilectus* sp.	Channel Islands	**0.12** (0.1–0.15)
	*Acanthascus* sp.	Channel Islands	**0.38** (0.32–0.47)
		Channel Islands	**0.50** (0.42–0.62)
	*Farreidae* sp.	Channel Islands	**0.56** (0.47–0.70)
	*Staurocalyptus* sp.	Channel Islands	**3.14** (2.62–3.92)
	Unknown species	Channel Islands	**2.77** (2.31–3.46)
	*Hexactinellida* sp.	Davidson Seamount	**0.16** (0.13–0.20)

The concentration uncertainty range, shown in parentheses, is calculated using ±1 SD from the average calibration factor.

Possible sources of superoxide within coral and sponge holobionts include the animals themselves, and their associated microbiomes (2). Although the impermeability of the cell membrane to superoxide should preclude photosynthetic endosymbionts from contributing to the extracellular superoxide pool, there have been observations of light-induced superoxide increases at the surfaces of shallow scleractinian corals (class Hexacorallia) (9). To reconcile these findings, researchers have proposed that algal endosymbionts provide the host with reactants and reduced enzymatic cofactors needed for superoxide production, such as oxygen and NADPH (2,10). Because the deep-sea corals and sponges studied here lack photosynthetic symbiotic algae, our results overturn this algal-centric paradigm that is generally accepted in shallow-water corals (9, 11). Instead, we provide definitive evidence that the activity of algal symbionts is not requisite for the production of extracellular superoxide within corals and sponges.

**Fig. 2.**
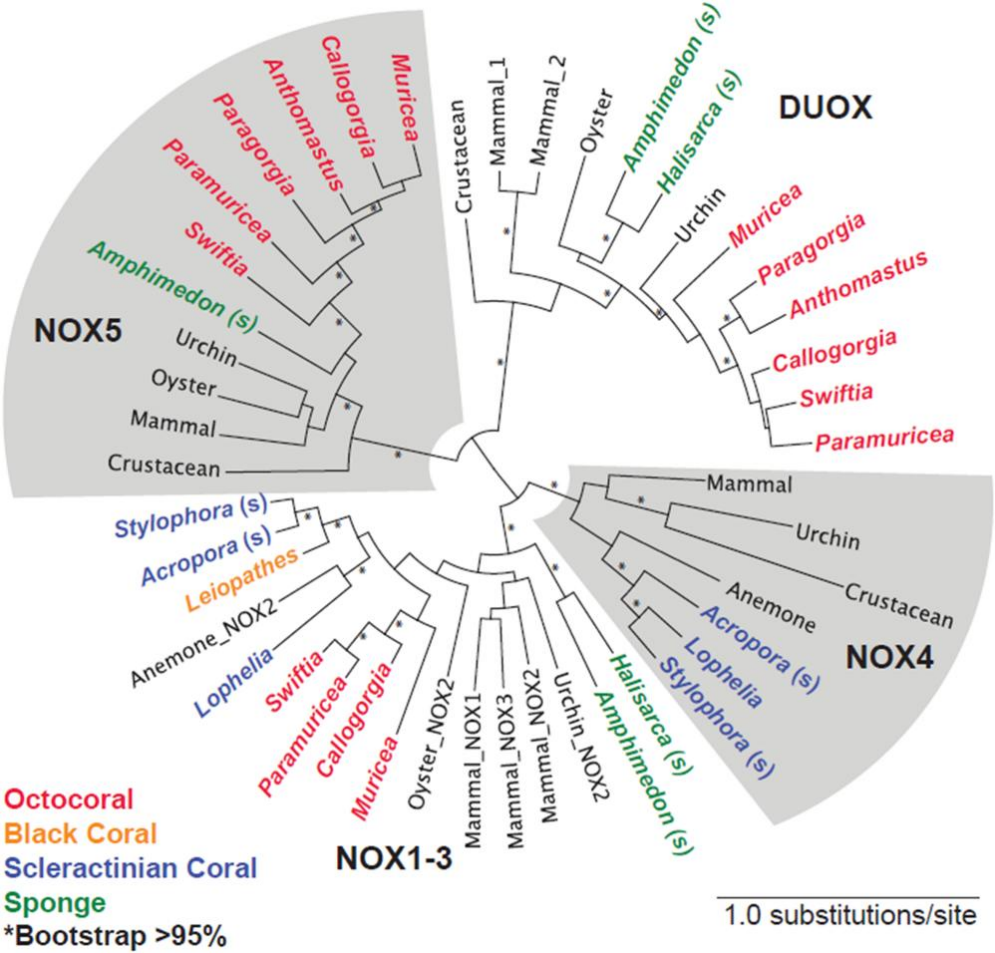
Maximum likelihood phylogenetic tree of NOX-like genes in corals and sponges, including reference animal genes. Coral and sponge groups are indicated with color fonts. All animals are deep sea or mesophotic, except shallow-water corals and sponges indicated with an (s). All sponges are from shallow water as no deep-sea genomes were available at the time of writing. The alternating shaded and nonshaded areas indicate the clades of NOX gene types.

Microbes hosted in the surface mucus layer of corals could be another source of superoxide. In fact, a range of marine bacterial heterotrophs is capable of extracellular superoxide production, leading to steady-state concentrations of ∼0.02 to 110 amol cell^−1^ h^−1^ (5, 6). However, a previous study showed that removal of the surface mucus layer did not change external superoxide concentrations associated with the colonies of *Porites astreoides* (2), suggesting that epibionts inhabiting the mucus layer of the coral are not the predominant source of surface-associated superoxide. Considering that a direct link between microbial community composition and extracellular superoxide levels has yet to be established, we suggest that the observed extracellular superoxide associated with the deep sea corals and sponges originates primarily from the animal component of the holobionts. Transmembrane NADPH oxidases (NOX), which generate ROS by coupling the oxidation of NADPH to the reduction of O_2_ at the cell surface, are widespread among eukaryotic organisms (12). Inhibition of NADPH oxidoreductase activity using the enzyme inhibitor diphenyleneiodonium eliminated extracellular superoxide production in a shallow scleractinian coral (9), implicating the activity of NOX enzymes in extracellular superoxide production. By surveying available deep-sea coral and sponge genomes and transcriptomes (including octocorals, black corals, and scleractinians), we find that NOX-like genes are, in fact, present within a diversity of species, including two genera measured for superoxide in this study (Figure 2). Thus, NOX genes may be widespread across the superclass Anthozoa and the Phylum Porifera, bestowing coral and sponge species with the genetic potential to produce extracellular superoxide. Targeting the controls on NOX expression in future investigations is needed to provide an improved understanding of the underlying physiological and ecological controls on superoxide production in these animals.

Production of ROS associated with corals and sponges may have important implications for understanding the physiology and ecology of deep-sea organisms, and the surrounding chemistry and redox state of the environment. For example, previous experiments have shown that the shallow-water scleractinian *Stylophora pistillata* produces the ROS hydrogen peroxide in response to the presence of prey and pathogens (13, 14). As sessile organisms, the ability of corals and sponges to alter the chemistry of their immediate surroundings via extracellular ROS production may be essential for their physiology, signaling, cellular defense, and the acquisition of carbon and micronutrient metals (15). Future research should focus on deciphering the controls on the production of superoxide and other ROS such as hydrogen peroxide, and the impacts of such ROS hotspots on the chemical ecology and biogeochemistry of deep-sea ecosystems.

## Materials and methods

### Superoxide measurements

We measured extracellular superoxide in situ using the submersible oceanic chemiluminescent analyzer of reactive intermediate species (SOLARIS), previously described in Taenzer et al. (8). For details on the calibration method, see the Supplementary material. We integrated SOLARIS onto *Alvin* and deployed it twice from the R/V *Atlantis* off the coast of California in October 2019 (cruise AT42-18), once at Davidson Seamount and once in the Channel Islands.

### Data collection

*Alvin*'s hydraulically powered manipulator, with 7° of movement and controlled by a spatially correspondent position feedback mechanism, was used to precisely position the tip of SOLARIS's sampling wand within a few centimeters or less of the organism's surface. SOLARIS's pump pulled seawater through the wand into the reaction cell. We operated SOLARIS from a custom graphical user interface within *Alvin*.

We collected ancillary data on the site geochemistry using a vehicle hosted conductivity-temperature-depth package and adjacent sensors (16). We also continuously collected data on superoxide concentrations in the seawater surrounding the animals, allowing for comparisons between animal surfaces and ambient background. Corals and sponges were collected in bioboxes and were taxonomically identified using morphological approaches (colony, polyp, and sclerite morphology) under light microscopy.

## Supplementary Material

pgad398_Supplementary_Data

## Data Availability

All the data are either given in the article and Supplementary material or available at 10.6084/m9.figshare.24002583.
