# Supplementary material for: Corals and sponges are hotspots of reactive oxygen species in the deep sea
Source: PNAS Nexus. 2023 Nov 15;2(11):pgad398. doi: 10.1093/pnasnexus/pgad398 (PMC10682969; doi:10.1093/pnasnexus/pgad398)
Supplement: pgad398_Supplementary_Data [file pgad398_supplementary_data.docx]

Supplementary Information for Corals and Sponges are Hotspots of Reactive Oxygen Species in the Deep-Sea

Lina Taenzer^1,2^, Scott D. Wankel^1^, Jason Kapit^3^, William A. Pardis^3^, Santiago Herrera^4^, Steven Auscavitch^5^, Kalina C. Grabb^1,2^, Erik Cordes^6^, Colleen M. Hansel^1*^

Correspondence to: chansel@whoi.edu

**Supplemental Materials and Methods**

*Superoxide Measurements*

Measurements of superoxide were made with the use of a recently developed submersible oceanic chemiluminescent analyzer of reactive intermediate species (SOLARIS), previously described in Taenzer et al., 2022. Briefly, SOLARIS is composed of two connected assemblies: the main body which holds reagents, and an analyzing assembly which consists of a sampling wand used to draw in seawater for analysis. In this study SOLARIS was integrated into the basket of the HOV ALVIN, and the manipulator arm was used to maneuver the sampling wand along the surfaces of organisms during dives. Dives for data collection were made near Davidson Seamount (35.76˚ N, -122.70˚W) and in the Channel Islands (33.66˚N, -119.19˚W).

SOLARIS is designed to quantify superoxide through the detection of the chemiluminescent signal generated upon the reaction of the probe methyl Cypridina luciferin analogue (MCLA) with superoxide in the respective sampling fluid. The reagents are pumped by peristaltic pumps from bags held on the main body of SOLARIS, as described in Taenzer et al., 2022. The signal is collected by a photomultiplier tube and can be followed in real-time from a graphical user interface which is also used to execute instrument controls (e.g., set pump speeds).

*Calibration*

SOLARIS is routinely calibrated in-situ using superoxide thermal source (SOTS-1) as a time dependent superoxide source, as described in Heller & Croot (2010) (16). In-situ calibrations allow for more accurate quantification of superoxide under varying temperature, and light conditions that may be encountered during a measurement period. At any specific time (t) the instantaneous concentration of superoxide in the SOTS-1 calibration solution (100 uM) is given by:

${[{O_{2}}^{-\cdot}]}_{i}=\sqrt{\frac{0.4k{[SOTS]}_{0}e^{-kt}}{2k_{D}}}$(Eq. 1)

where k is the temperature-dependent decay constant, k, for SOTS-1 and k_D_ is the uncatalyzed second-order dismutation rate constant. A calibration sequence compares the background signal produced by the reaction of MCLA (6.3 mL/min) with reagent background seawater (6.3 mL/min), with that produced by doping in the SOTS-1 solution at (1.5 mL/min). Tother, the steady state signal of the SOTS-1 solution, and knowledge of the time-dependent superoxide concentration (given by Eq. 1) are used to establish a calibration factor (photon count/concentration of superoxide).

*Deep-sea coral genomes and transcriptomes*

Genomic DNA sequences were obtained for the deep-sea and mesophotic octocorals *Paragorgia arborea* from Davidson Seamount*,* the octocorals *Muricea pendula, Swiftia exserta,* and *Anthomastus sp.* and the black coral *Leiopathes glaberrima* from the Gulf of Mexico*,* and the deep-sea scleractinian *Lophelia pertusa* from the southeastern USA (17)*.* For *Paragorgia arborea, Muricea pendula, Swiftia exserta, Anthomastus sp.,* and *Lophelia pertusa* genomic DNA was purified using a salting-out protocol (*18*). Except for *Anthomastus,* the DNA of these corals was used to construct single molecule real-time (SMRT) libraries and generate sequences on a PacBio Sequel II machine. Sequence reads were assembled using flye v2.9 (*19*), followed by polishing with NextPolish v1.3.1 (*20)* and the haplotig and contig overlap removal program purge_dups v.1.2.3 (*21*). Genes and translations were predicted using the pipeline FunAnnotate v.1.8.9 (*22*). DNA sequences for *Anthomastus* sp. was produced using an Illumina NovaSeq instrument (paired-end, 150bp). Raw DNA sequence data (Illumina paired-end, 100bp and 250bp) from *Leiopathes glaberrima* were retrieved from NCBI’s SRA Accession SRX6913634 (*23*). Reads were quality filtered and trimmed using Trimmomatic (*24*). Sequence reads were assembled using SPAdes (*25*). Genes and translations were predicted using the pipeline FunAnnotate v.1.8.9 (*22*). Published transcriptome assemblies were retrieved for the deep-sea octocorals *Paramuricea biscaya (26)* and *Callogorgia delta (27).*

*NOX-like genes in deep-sea corals and sponges*

To search for NOX/DUOX-like genes, Hidden Markov models (HMMs) were created from alignments of published amino acid sequences for each NOX gene type (*27*, *28*). These models were queried against aminoacidic sequences predicted from genome or transcriptome assemblies of deep-sea corals (Additional Datafile) using HMMER v3.3 (*29)*. In a few instances, protein sequences were fragmented. In these cases, contigs were merged if they contained overlapping sequences (Additional Datafile). Genome and transcriptome data represented closely related species (i.e., same species, genus, or family) to those measured *in situ*.

We identified protein domains for each amino acid sequence that yielded a significant match to a NOX gene type HMM, using InterProtScan (*30)* in Geneious Prime v2022.2.2 for. Significant matches in HMMER were defined as a full sequence E-value <1e-20 and score >200. The most significant matches were queried against NCIB’s non-redundant protein sequence database (nr) using blastp (BLAST ^®^). Annotated amino acid sequences were aligned using Clustal Omega (*31*). We included published NOX gene sequences of sponges and reference animals, plants, and fungi (Table S6).

Amino acid sequence alignments and annotations were visually inspected in Geneious. Sequences that contained Ferric Reductase, FAD binding 8, and NAD binding 6 domains were identified as putative NOX-like genes. NOX-like genes that contained EF-hands upstream were classified as putative NOX5-like genes. NOX-like genes that contained Animal Peroxidase (Heme-dependent peroxidase) and EF-hand domains upstream were classified as putative DUOX-like genes.

Putative NOX-like genes were further classified by 1) sequence alignment incorporating reference eukaryotic amino acid sequences published by Gandara et al (2017) using Clustal Omega (*31)*, and 2) Neighbor-Joining clustering (Jukes-Cantor distance model) using the Geneious Tree Builder. Phylogenetic trees were inferred using Maximum Likelihood in RAxML v.8.2.11 (*32)* (GAMMA BLOSUM62 protein model; rapid hill-climbing). Statistical support for inferred clades was assessed through bootstrapping (200 replicates).

We found NOX-like genes type NOX2 (Additional Datafile), NOX5 (Additional Datafile), and DUOX (Additional Datafile) in deep-sea and mesophotic octocoral genomes (Additional Datafile). These genes are present in the transcriptomes of *Paramuricea* type B3 and *Callogorgia delta (27)*, thus are likely constitutively expressed. This indicates that the Class Octocorallia has a functional set of NOX genes NOX2, NOX5 and DUOX.

We found NOX-like genes type NOX2 (Additional Datafile) and NOX4 (Additional Datafile) in deep-sea scleractinians and black coral genomes (Additional Datafile). We found Animal Peroxidase (Heme-dependent peroxidase) genes with high homology to the DUOX domain, but no full DUOX genes. These genes are also found in the transcriptome of *Lophelia pertusa (33).* Similarly, we found NOX2-like, NOX4-like, and Animal Peroxidase-like genes in published sequence datasets of shallow-water scleractinians and anemones. This indicates that the Class Hexacorallia has a functional set of NOX genes NOX2 and NOX4, and of Animal Peroxidases.

Published genome sequence data indicate that shallow water sponges have NOX-like genes type NOX2, NOX5, and DUOX (Additional Datafile). No data from deep-sea sponges were available.

**Supplementary References**

1. Heller, M. I., & Croot, P. L. (2010). Superoxide Decay Kinetics in the Southern Ocean. *Environmental Science & Technology*, *44*(1), 191–196. <https://doi.org/10.1021/es901766r>
2. Herrera, S., & Cordes, E. E. (2023). Genome assembly of the deep-sea coral Lophelia pertusa. *GigaByte (Hong Kong, China)*, *2023*, gigabyte78. https://doi.org/10.46471/gigabyte.78
3. Herrera, S. (2021). *Salting-out protocol for extracting HMW genomic DNA from frozen octocorals"* [Data set]. https://doi.org/10.17504/protocols.io.bypypvpw
4. Kolmogorov, M., Yuan, J., Lin, Y., & Pevzner, P. A. (2019). Assembly of long, error-prone reads using repeat graphs. *Nature Biotechnology*, *37*(5), 540–546.
5. Hu, J., Fan, J., Sun, Z., & Liu, S. (2020). NextPolish: a fast and efficient genome polishing tool for long-read assembly. *Bioinformatics* , *36*(7), 2253–2255.
6. Guan, D., McCarthy, S. A., Wood, J., Howe, K., Wang, Y., & Durbin, R. (2020). Identifying and removing haplotypic duplication in primary genome assemblies. *Bioinformatics* , *36*(9), 2896–2898.
7. Palmer, J. M. (2016). *Funannotate: a fungal genome annotation and comparative genomics pipeline* (1.8.9) [Computer software]. https://github.com/nextgenusfs/funannotate
8. Vohsen, S. A., Anderson, K. E., Gade, A. M., Gruber-Vodicka, H. R., Dannenberg, R. P., Osman, E. O., Dubilier, N., Fisher, C. R., & Baums, I. B. (2020). Deep-sea corals provide new insight into the ecology, evolution, and the role of plastids in widespread apicomplexan symbionts of anthozoans. *Microbiome*, *8*(1), 34.
9. Bolger, A. M., Lohse, M., & Usadel, B. (2014). Trimmomatic: a flexible trimmer for Illumina sequence data. *Bioinformatics* , *30*(15), 2114–2120.
10. Prjibelski, A., Antipov, D., Meleshko, D., Lapidus, A., & Korobeynikov, A. (2020). Using SPAdes DE Novo Assembler. *Current Protocols in Bioinformatics / Editoral Board, Andreas D. Baxevanis ... [et Al.]*, *70*(1), e102.
11. DeLeo, D. M., Herrera, S., Lengyel, S. D., Quattrini, A. M., Kulathinal, R. J., & Cordes, E. E. (2018). Gene expression profiling reveals deep-sea coral response to the Deepwater Horizon oil spill. *Molecular Ecology*, *27*(20), 4066–4077.
12. DeLeo, D. M., Glazier, A., Herrera, S., Barkman, A., & Cordes, E. E. (2021). Transcriptomic responses of deep-sea corals experimentally exposed to crude oil and dispersant. *Frontiers in Marine Science*, *8*. <https://doi.org/10.3389/fmars.2021.649909>
13. Kawahara, T., Quinn, M. T., & Lambeth, J. D. (2007). Molecular evolution of the reactive oxygen-generating NADPH oxidase (Nox/Duox) family of enzymes. *BMC Evolutionary Biology*, *7*(1), 109. <https://doi.org/10.1186/1471-2148-7-109>
14. Finn, R. D., Clements, J., & Eddy, S. R. (2011). HMMER web server: interactive sequence similarity searching. *Nucleic Acids Research*, *39*(Web Server issue), W29-37.
15. Mitchell, A. L., Attwood, T. K., Babbitt, P. C., Blum, M., Bork, P., Bridge, A., Brown, S. D., Chang, H.-Y., El-Gebali, S., Fraser, M. I., Gough, J., Haft, D. R., Huang, H., Letunic, I., Lopez, R., Luciani, A., Madeira, F., Marchler-Bauer, A., Mi, H., … Finn, R. D. (2019). InterPro in 2019: improving coverage, classification and access to protein sequence annotations. *Nucleic Acids Research*, *47*(D1), D351–D360.
16. Sievers, F., Wilm, A., Dineen, D., Gibson, T. J., Karplus, K., Li, W., Lopez, R., McWilliam, H., Remmert, M., Söding, J., Thompson, J. D., & Higgins, D. G. (2011). Fast, scalable generation of high-quality protein multiple sequence alignments using Clustal Omega. *Molecular Systems Biology*, *7*, 539.
17. Stamatakis, A. (2014). RAxML version 8: a tool for phylogenetic analysis and post-analysis of large phylogenies. *Bioinformatics* , *30*(9), 1312–1313.
18. Glazier, A., Herrera, S., Weinnig, A., Kurman, M., Gómez, C. E., & Cordes, E. (2020). Regulation of ion transport and energy metabolism enables certain coral genotypes to maintain calcification under experimental ocean acidification. *Molecular Ecology*, *29*(9), 1657–1673.
